# LONELINESS, SOCIAL ISOLATION, AND HEALTH WITHIN THE CONTEXT OF A GLOBAL PANDEMIC

**DOI:** 10.1093/geroni/igad104.0259

**Published:** 2023-12-21

**Authors:** Eileen Graham, Shevaun Neupert, Toni Antonucci

## Abstract

Social connectivity is a crucial human experience. The objective experience of being disconnected (i.e., social isolation) and the subjective feeling of being disconnected (i.e., loneliness) are related but distinct experiences that have unique impacts on physical, mental, and cognitive health. The COVID-19 pandemic forced much of the global population into a prolonged state of isolation, and many older adults found themselves abruptly cut-off from important sources of social interaction. The lasting impacts of the pandemic are still unknown. This symposium brings together several projects addressing key questions about longitudinal processes of loneliness and social isolation among middle aged and older adults, both prior to and during the COVID-19 pandemic. Jackson will present results from a coordinated data analysis (CDA) showing the extent to which loneliness, social isolation, and social asymmetry are related to MCI, dementia, mortality, and life expectancy. Rule will discuss the impact of stroke on trajectories of loneliness in a CDA. Zhang and Fingerman will present findings that show how loneliness and social isolation vary among individuals with functional limitations. Zavala will present results from a CDA that explored daily patterns of loneliness during the early months of the COVID-19 pandemic. Lastly, Beck will discuss a CDA that examined trajectories of social asymmetry prior to and during the COVID-19 pandemic. Antonucci will synthesize and discuss each project within the context of broader implications for empowering older adults. In sum, this symposium presents novel evidence showing that loneliness and social isolation in older adulthood are impactful predictors of health.

